# Incidence and Risk Factors for Acute Kidney Injury during the Treatment of Methicillin-Sensitive *Staphylococcus aureus* Infections with Cloxacillin Based Antibiotic Regimens: A French Retrospective Study

**DOI:** 10.3390/jcm10122603

**Published:** 2021-06-12

**Authors:** Romain Crochette, Camille Ravaiau, Lucia Perez, Jean-Philippe Coindre, Giorgina Barbara Piccoli, Sophie Blanchi

**Affiliations:** 1 Department of Nephrology, Le Mans General Hospital, 72000 Le Mans, France; camille_ravaiau@hotmail.fr (C.R.); jpcoindre@ch-lemans.fr (J.-P.C.); gpiccoli@ch-lemans.fr (G.B.P.); 2 Department of Infectious Diseases, Le Mans General Hospital, 72000 Le Mans, France; lperez@ch-lemans.fr (L.P.); sblanchi@ch-lemans.fr (S.B.); 3 Dipartimento di Scienze Cliniche e Biologiche, Università di Torino, 10124 Torino, Italy

**Keywords:** Staphylococcus infection, cloxacillin, acute kidney injury, diuretics, endocarditis

## Abstract

Background: Cloxacillin has been associated with the occurrence of acute kidney injury (AKI). The incidence of this complication in the literature is low (2.5–3.5%) and probably underestimated, since most studies were done by selecting the presence of AKI in discharge codes. Objectives: The primary goal was to define the incidence of AKI in patients with a methicillin-sensitive *Staphylococcus aureus* infection treated with cloxacillin based antibiotic regimens. The secondary goals were to identify the risk factors associated with this complication and to describe the characteristics of AKI. Patients and methods: We carried out a retrospective study. The inclusion criteria were adult patients hospitalized in a medical department at the Le Mans Hospital between 1 July 2012 and 1 July 2019 with a diagnosis of methicillin-sensitive *Staphylococcus aureus* infection treated with cloxacillin. Results: One hundred twenty-three patients were included in the study. Forty-two patients (34.2%) developed AKI. In the multivariate analysis, age, the use of diuretics and the presence of endocarditis were independently associated with AKI. Age was associated with an OR of 4.38 (*p* = 0.002) for patients older than 75, being treated with diuretics was associated with an OR of 2.94 (*p* = 0.036) for loop diuretics and an OR of 3.05 (*p* = 0.027) for non-loop diuretics; type of infection was associated with an OR of 3.42 (*p* = 0.012) for endocarditis. Conclusions: The occurrence of AKI is frequent during cloxacillin based antibiotic regimens for methicillin-sensitive *Staphylococcus aureus* infections. Being older than 75, being treated with diuretics and the presence of endocarditis were the main risk factors for AKI in our population.

## 1. Introduction

Cloxacillin, an antibiotic in the penicillin family, is recommended for treating severe methicillin-sensitive *Staphylococcus aureus* infections, such as bacterial endocarditis [1] and osteoarticular infections [2].

Treatment of these infections requires high doses of cloxacillin in order to achieve a time above minimum inhibitory concentration (MIC) > 50%. The French National Agency for Medicines and Health Products (ANSM) reviewed the marketing authorization (MA) for cloxacillin in 2011 and authorized the use of doses ranging from 8 g to 12 g per day in four to six injections. These doses are higher than those originally indicated in the summary of product characteristics (SPC) (maximum 6 g per day) [3].

The SPC does not recommend adjusting the doses for patients who have an estimated glomerular filtration rate (eGFR) above 30 mL/min. Reducing the dosage by 50% for patients who have an eGFR < 30 mL/min is recommended. French recommendations also consider the option of continuous administration of cloxacillin [4]. The blood and tissue concentration of this antibiotic depends on treatment schedule (continuous versus discontinuous) and kidney function [5,6].

Cloxacillin, and other molecules belonging to the same family of isoxazolyl penicillins (flucloxacillin, dicloxacillin), have been associated with the occurrence of acute kidney injury (AKI), regardless of whether they are used alone or in combination with other antibiotics, such as aminoglycosides [7,8,9,10]. As for other beta lactam antibiotics, combination therapies with vancomycin may increase the risk for AKI [11].

In patients treated with cloxacillin, AKI is reported to be secondary to acute immuno-allergic interstitial nephritis or acute tubular necrosis [8,10,12,13,14,15,16]. The incidence of this complication in the literature is, however, low (2.5%–3.5%) [10,17], and some studies suggest that it is probably underestimated [10].

It was in this context that we carried out our study, whose primary goal was to define the incidence of AKI in patients with a methicillin-sensitive *Staphylococcus aureus* infection treated with cloxacillin. The secondary goals were to identify the risk factors associated with this complication and to describe the characteristics of AKI.

## 2. Methods

### 2.1. Study Design

We conducted a retrospective study of patients who were hospitalized at the Centre Hospitalier le Mans (CH Le Mans) between 1 July 2012 and 1 July 2019.

### 2.2. Study Setting

The CH le Mans is one of the largest non-university hospitals in France. The study involved 14 units (about 300 beds) in the hospital’s medicine wards.

### 2.3. Participants and Data

The inclusion criteria were adult patients hospitalized in a medicine ward at the CH Le Mans between 1 July 2012 and 1 July 2019 with a diagnosis of methicillin-sensitive *Staphylococcus aureus* infection treated with cloxacillin.

The exclusion criteria were minors (person under the age of 18), patients on chronic dialysis, and patients hospitalized in the ICU (Intensive Care Units). This choice was motivated by the fact that the hemodynamic instability of ICU patients may be a further confounder for AKI development.

Patients were identified by running a search in the PMSI (Program for the Medicalization of Information Systems) database at CH Le Mans using the broadest ICD-10 code for identification of the infection (staphylococcus infection A410). The PMSI provides a synthetic and standardized description of the medical activity of health care institutions. All clinical charts were reviewed: only cases with a methicillin-sensitive *Staphylococcus aureus* (MSSA) infection, treated with cloxacillin for more than one day, were selected for the present analysis.

Data were collected from medical charts. All the clinical charts (paper up to 2015 and electronic thereafter) were retrieved and reviewed for data extraction by the authors Romain Crochette and Camille Ravaiau; controversial cases were reviewed by the author Sophie Blanchi.

No statistical power calculation was made since the aim of the study was merely to describe the occurrence of AKI in the setting of cloxacillin use and to identify eventual clinical elements associated with AKI.

### 2.4. Study Endpoints and Definitions

The primary objective of the study was to define the incidence of AKI in patients with methicillin-sensitive *Staphylococcus aureus* infection treated with cloxacillin.

AKI was defined according to the RIFLE criteria based on serum creatinine (Screat) only, since urine outputs were not available. RIFLE defines three grades of increasing severity of AKI: risk (class R) defined by an increased Screat × 1.5 or GFR decrease > 25 % from baseline, injury (class I) defined by an increased Screat × 2 or GFR decrease > 50% from baseline and failure (class F) defined by an increased Screat × 3 or GFR decrease > 75% from baseline or a S creat > 4 mg/dL (350 µmol/L) and two outcome classes (loss and end-stage kidney disease) [18]. When baseline serum creatinine (within 6 months before hospitalization) was unavailable, the creatinine level on admission was considered as a baseline value.

The secondary objectives were to identify the risk factors that were independently associated with AKI and to describe the characteristics of patients with AKI. Potential risk factors analyzed were:

- Age.
- Body Mass Index (BMI).
- Chronic Kidney Disease (CKD). CKD was defined by an eGFR < 60 mL/min/1.73 m^2^ [19] at baseline according to the MDRD equation (without race adjustment, since race was not recorded in medical charts).
- Comorbidities (diabetes, hypertension, Charlson comorbidity index).
- Smoking.
- High alcohol consumption. It was defined as any alcohol use described as excessive or pathological in clinical charts.
- Serum albumin (g/L).
- Hypotension during hospitalization: defined by at least one episode of acute hypotension (mean arterial pressure of <65 mmHg or a systolic arterial pressure of <100 mmHg) recorded in the patient’s medical file.
- Nephrotoxic drugs: Proton Pump Inhibitors (PPI), Nonsteroidal Anti-Inflammatory Drugs (NSAID), Renin Angiotensin Aldosteron System (RAAS) inhibitors, loop diuretics, non-loop diuretics, statins.
- Other antibiotics.
- Iodinated contrast media.
- Type of Infection (endocarditis vs. other infections: bacteremia, catheter blood stream infection, bones infection, and urinary tract infection).
- Regarding cloxacillin use:
  - Dose (g per day).
  - Mode of administration (IV, versus oral).
  - Infusion type (continuous versus intermittent).
  - Treatment duration.

### 2.5. Statistical Analysis

The qualitative variables were presented as figures and percentages. The quantitative variables were presented as median + Inter Quartile Range, the maximum and the minimum, or as mean + standard deviation.

The characteristics of patients who developed AKI were compared with those of patients who did not present AKI. Depending on their distribution, the quantitative variables were compared using parametric tests (Student’s *t*-test) or non-parametric tests (Mann–Whitney test). Age, dose of cloxacillin per day, duration of cloxacillin administration, and length of hospitalization were found to have a non-parametric distribution. The comparison of categorical variables was performed using Chi-squared or Fisher’s tests. As the relation between AKI and age was nonlinear, we analyzed this factor as a categorial variable.

In order to determine the factors associated with AKI, we first explored the potential associations by univariate logistic regression. Then, we built a multivariate model entering the variables associated with AKI with a significance level *p* < 0.10 in the univariate analysis and the ones strongly associated with AKI in the medical literature, after testing for collinearity.

The added variable was CKD, due to its clinical relevance as a recognized risk factor for AKI (19).

Age was dichotomized at the median.

The final model was developed through a backward stepwise method.

Residuals of the multiple regression were verified. Calibration of the model was assessed by a Hosmer–Lemeshow goodness-of-fit method. Discrimination of the model was assessed by the area under the Receiver Operating Characteristic (ROC) curve method. (supplementary data, Figures S1 and S2).

All of the tests were two-tailed. A *p*-value < 0.05 was considered statistically significant.

Statistical analyses were performed with StataCorp. 13 (Stata Statistical Software: Release 13. StataCorp LP., College Station, TX, USA), R (R Core Team, 2021) and Excel 2007 (Microsoft Corporation, Redmond, WA, USA) software.

### 2.6. Ethical Issues

The study was approved by the ethics committee at CH Le Mans on 7 November 2019. An information letter was sent to all patients or, in case of death, to the family members whenever identifiable. Patients and family had the right to refuse access and to rectify personal data in keeping with French legislation.

## 3. Results

### 3.1. Baseline Data and Data at Diagnosis of AKI

**Figure 1:**
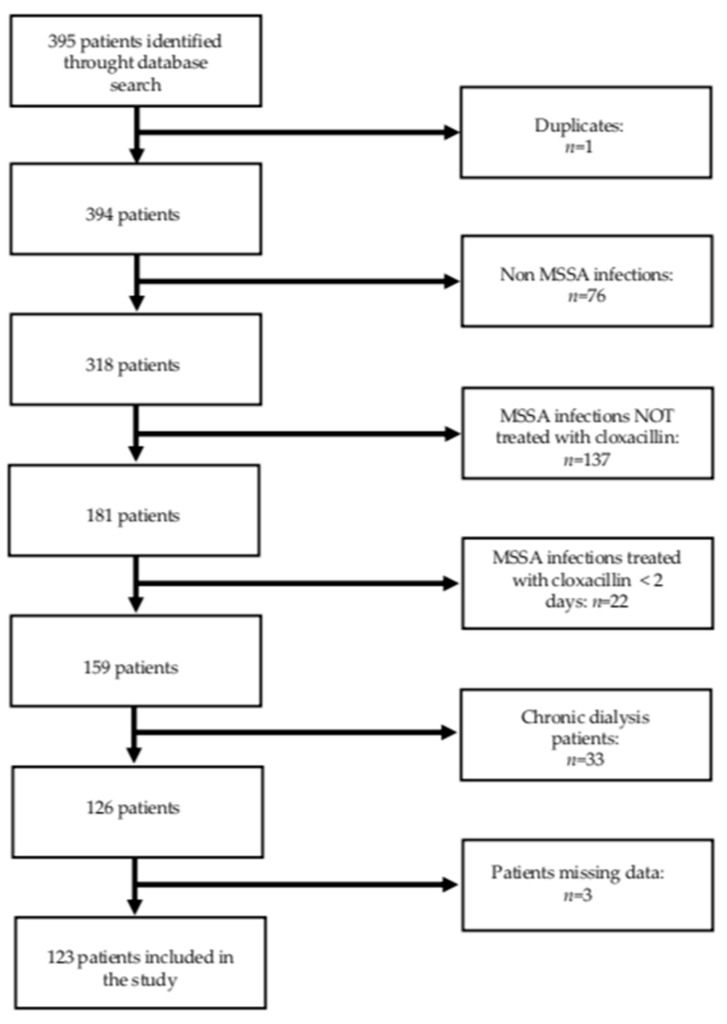
Study flow chart.

After reviewing the clinical charts of all patients (395) recorded in the hospital’s discharge data base as having a staphylococcus infection, 123 patients were included in the study (Figure 1). Data were missing in only three cases.

Forty-two patients (34.2%) developed AKI. According to the RIFLE classification, at diagnosis, 19 patients were in the RISK category, 11 in INJURY, and 12 in FAILURE [18].

In a context of a predominantly male, relatively old population, at high comorbidity (median Charlson score of 6), patients with AKI were significantly older, without other major baseline differences.

As for treatments, AKI was more frequently associated with diuretic use assessed at first AKI diagnosis, while the association with RAAS inhibitors was not significant.

Type of infection was also associated with AKI. Conversely, the cumulative dose of cloxacillin received up to the diagnosis of AKI was not different in the AKI versus non-AKI groups. Treatment duration was not different in the two groups nor was the use of continuous or intermittent schedules.

The presence of AKI had a significant impact on hospital stay (27 days versus 19 days, *p* = 0.0059) while the increase in in-hospital mortality (28.6% versus 14.8%, *p* = 0.093) did not reach statistical significance (Table 1). However, patients who were in the INJURY and FAILURE categories had a significantly higher mortality rate than patients in the RISK category, compared to patients without AKI (34.8% versus 16%, *p* = 0.04).

### 3.2. Clinical Data at AKI Presentation

Forty-two patients were classified as having AKI. Only one patient was biopsied and was diagnosed with IgA nephropathy. The characteristics of the patients who developed AKI are described in Table 2. The median creatinine level at baseline was 93.5 µmol/L (45–183) reaching a peak of 221 µmol/L (95–1006). Six patients needed dialysis. Four patients presented a cutaneous reaction, 4 patients developed an encephalopathy, 13 developed hepatitis, and 4 had eosinophilia. At diagnosis, the median proteinuria was 0.52 g/L (0–4.34), the median proteinuria/creatininuria ratio (PCR) was 0.64 g/g (0–5.31), but data were missing for 17 patients. Hematuria was present in nine patients and leukocyturia in 18 patients (Table 2).

The median creatinine level at hospital discharge was 140 µmol/L (49–429).

The average dose of cloxacillin received was not different between the categories in the RIFLE classification (*p* = 0.798).

Of the 47 patients presenting with CKD (defined by an eGFR < 60 mL/min/1.73 m^2^ at baseline), 35 had an eGFR ≥ 30 mL/min/1.73 m^2^ (no dose adjustment). Nineteen received a daily dose of 12 g of cloxacillin and nine developed AKI. Thirteen received a daily cloxacillin dose of between 8 g and 10 g and six developed AKI. Three received a daily cloxacillin dose of 6 g and all developed AKI. Twelve patients had an eGFR < 30 mL/minute/1.73 m^2^. Three received a daily cloxacillin dose of between 6 g and 12 g and one developed AKI. Nine received a daily dose ≤ 6 g of cloxacillin and only one developed AKI.

Cloxacillin plasma concentrations were measured in only 8 of 123 patients. The only patient without AKI, and a CKD, had a plasma concentration < 50 μg/mL. In the seven patients with AKI, five had a plasma concentration > 50 μg/mL (83.7–362 μg/mL) at the moment when AKI was diagnosed.

### 3.3. Risk Factors for AKI

The variables associated with AKI with a significance level *p* < 0.10 in the univariate analysis were: age, RAAS inhibitors, loop diuretics, non-loop diuretics, vancomycin use, aminoglycoside use, type of infection, and hypotension (Table 3). Age was dichotomized at the median (≤75 years and >75 years). Type of infection was categorized as endocarditis vs. other infections.

In the univariate analysis, the factors significantly associated with AKI were: being older than 75 years of age, with an OR = 3.42 (*p* = 0.0017); being treated with diuretics, with an OR = 2.77 (*p* = 0.0093) for loop diuretics and an OR = 2.44 (*p* = 0.0383) for non-loop diuretics; and having infectious endocarditis, with an OR of 4.31 (*p* = 0.0008). Conversely, the presence of a known CKD was not confirmed as a significant risk factor in this cohort.

In the multivariate analysis, in the model built with all the variables previously resulting with a *p*-value <0.10 and CKD, due to its usual relevance in favoring the development of AKI, age, use of diuretics and the type of infection were independently associated with AKI. Age was associated with an OR of 4.38 (*p* = 0.002) for patients older than 75, being treated with diuretics was confirmed as statistically significant with an OR of 2.94 (*p* = 0.036) for loop diuretics and an OR of 3.05 (*p* = 0.027) for non-loop diuretics. There was strong evidence that the type of infection was associated with the odds of AKI and the OR for endocarditis was 3.42 (*p* = 0.012) (Table 4).

## 4. Discussion

The first result of interest in our study is the high frequency of AKI in patients treated with cloxacillin for MSSA infection, as 42 patients, 34.2% of the population, developed this complication. This incidence is considerably higher than what was reported in previous studies [10,17]. Differences in the diagnostic criteria, identification of the patients, and baseline characteristics of the population may account for the important differences in incidence. In particular, in the study by Khalili et al. [17], out of 75 patients who received cloxacillin, only six were diagnosed with AKI (8%). However, patients were younger (median age 46.5 years) and were hospitalized only in an infectious disease unit. Patients with CKD, requiring dialysis in the first 48 h of hospitalization, receiving iodinated contrast media and other nephrotoxic treatments were excluded. In the study by Lavergne et al. [10], the cumulative risk of AKI was 3.4% per patient treated with cloxacillin. However, patients who developed AKI, while treated with cloxacillin, were identified via the pharmacovigilance center, passive surveillance, and a PMSI database query using ICD-10 codes suggesting drug-induced kidney injury. In our study, patients were included according to PMSI search with the broadest ICD-10 code “staphylococcus infection”. The files were then reviewed to determine whether the patients, with a methicillin-sensitive *Staphylococcus aureus* (MSSA) infection treated with cloxacillin, had presented an AKI during course hospitalization, and the sensitive RIFLE classification was employed [18]. To highlight the importance of patient selection criteria, in our study for 17/42 patients (40%), AKI was not mentioned in the PMSI codes. We can therefore hypothesize that, in the studies that used post-marketing surveillance reports, the frequency of AKI was underestimated [10], as probably only the most serious cases were reported. Consistent with this interpretation, 45% of the patients with AKI in our study were in the RIFLE RISK category.

The relatively low awareness of AKI is also reflected in the lack of timely laboratory tests. In fact, in our series, 40.5% of patients did not have a proteinuria/creatinuria ratio (PCR) measured and 25% of the patients did not have a urinary cytobacterial test at time of diagnosis (Table 2). Furthermore, antimicrobial drug level monitoring was seldom performed.

The physiopathology of AKI after cloxacillin use is only partially known, due also to the paucity of kidney biopsy data in this context. For the beta-lactams family, nephrotoxicity is considered as due to tubular cell toxicity or interstitial inflammation. In that setting, it has been reported that tubular toxicity is driven by three main mechanisms: interference with tubular transport, impaired mitochondrial function, and increased oxidative stress [20].

In our series, in keeping with the literature [21], AKI was associated with a significantly longer duration of hospitalization (Table 1).

The risk factors of AKI previously identified in patients treated by cloxacillin were older age and treatment with well-known nephrotoxic drugs (PPI, NSAID, diuretics, RAAS inhibitors, gentamicin). Hemodynamic instability was frequent in the previous reports and several patients received iodinated contrast media [10].

Our study identified only age, type of infection, and treatment with diuretics as factors that are independently associated with the occurrence of AKI in patients treated with cloxacillin for an MSSA infection.

The relationship with age is not surprising: Staphylococcus infections are common in older patients [22]. Furthermore, age is a risk factor for AKI during sepsis [21]. Identifying age >75 years as a risk factor for AKI during cloxacillin therapy clearly indicates that more attention needs to be paid to cloxacillin plasma concentrations and kidney function monitoring in this population.

Diuretics were the only treatment linked to AKI in our study (OR 2.94 for loop diuretics and OR 3.05 for non-loop diuretics). While retrospective studies can only describe associations, the hypothesis is that diuretics can cause or aggravate hypovolemia [23], leading to renal microcirculatory dysfunction, which is one of the cornerstones of AKI physiopathology [24], in particular in the presence of tubular toxicity, which is the main pathogenetic mechanism in the case of cloxacillin-induced AKI [10,20].

AKI was significantly more frequent in cases of endocarditis, a finding in line with both the presence of a severe disease and higher comorbidity.

Conversely, in our study, other known nephrotoxic drugs, including RAAS inhibitors, NSAID, and PPI, were not associated with this complication. In the literature, RAAS inhibitors have been linked to AKI [23], although a beneficial effect has been described in sepsis, associated with decreased 30-day mortality [25]. Furthermore, in our study, the co-administration of glycopeptides, aminoglycosides, and other penicillins was not associated with AKI (Table 1), as it has been in other reports [7,8,9,10]. This may be explained by the fact that our teams followed the recent recommendations on treatment of MSSA endocarditis, shortening the duration of aminoglycoside prescription [1]. However, the cumulative doses and the blood levels of these antibiotics were not analyzed in this study; this limitation may be overcome in the future by a higher appreciation of the risk of AKI in this context, allowing also easier comparisons among settings. Furthermore, there was no correlation between the average dose of cloxacillin received and the severity of AKI.

Pre-existing chronic kidney disease was not associated with AKI. However, not adapting the dosage of cloxacillin for patients with eGFR < 30 mL/min/1.73 m^2^, as recommended, appears to be a risk factor. Of the 12 patients with eGFR < 30 mL/min/1.73 m^2^, 1 of the 3 who received a daily cloxacillin dose between 6 g and 12 g developed AKI compared to 1 of the 9 who received a daily dose ≤ 6 g.

This study has a number of limitations. First of all, it is a retrospective study, based on patients’ medical files. This may have caused some biases: for example, in the case of a lack of baseline creatinine level, we used the one recorded when the patient was admitted; in severely ill patients, this may have led to an underestimation of the number with AKI and to an overestimation of the number with CKD. Secondly, the non-comparative nature of the study means it is not possible to directly attribute AKI to cloxacillin treatment. We tried to stratify our analysis according to the type of infection (endocarditis vs. other infections) and according to the treatment received (cloxacillin alone vs. cloxacillin in combination with other antibiotics). However, the low numbers of cases did not allow further statistical analysis (only 30/123 patients had endocarditis and only 27/123 patients received Cloxacillin alone). Furthermore, we did not include patients who were hospitalized in intensive care units, as these patients usually present multiple risk factors for AKI, which would have made it difficult to determine the role of cloxacillin. Bleeding and surgery were not collected and may be therefore unrecognized risk factors for AKI. Finally, serum creatinine is an imperfect biomarker for AKI, especially in septic patients [26]. However, urinary biomarkers such as Kidney Injury Molecule-1 or Neutrophil Gelatinase-associated Lipocalin which are more sensitive markers for early diagnosis of AKI are not measured routinely in our hospital.

The results of our study therefore need to be confirmed by a prospective randomized study comparing two recommended treatments for methicillin-sensitive *Staphylococcus aureus* infections, such as cloxacillin and cefazolin.

The strength of his study is that it shows that AKI occurs more frequently in patients treated with cloxacillin for a methicillin-sensitive *Staphylococcus aureus* infection and that it identifies risk factors for AKI in this population. Besides age, treatment with diuretics is a potentially modifiable risk factor and the importance of dose adaptation in severe chronic kidney disease must be emphasized.

## 5. Conclusions

The occurrence of AKI is frequent during cloxacillin treatment for methicillin-sensitive *Staphylococcus aureus* infections. AKI, associated with longer hospital stay and possibly with death, is probably under-reported in discharge charts. Being older than 75, the presence of endocarditis and being treated with diuretics are the main non-modifiable and modifiable risk factors for AKI in our population. Randomized controlled trials comparing cloxacillin with other antibiotics are needed to better understand the role of treatment and baseline conditions in determining this potentially severe complication.

## Figures and Tables

**Table 1:** Characteristics of patients included in the CLOX-AKI study, CH Le Mans, 1 July 2012 to 1 July 2019.

	Total = 123	AKI Group = 42	Non-AKI Group = 81	*p*
Gender *n* (%)				0.224
Male	85 (69.1)	26 (61.9)	59 (72.8)	
Age *n* (%)				0.002
≤75 years	62 (50.4)	13 (31)	49 (60.5)	
>75 years	61 (49.6)	29 (69)	32 (39.5)	
Median age (IQR) (min-max) *	75 (61–84) (36–101)	81 (69–88) (51–96)	70 (60–82) (36–101)	0.0007
Mean BMI (std) **	26.5 (5.9)	26.5 (6.4)	26.1 (5.5)	0.2202
CKD *n* (%)	47 (38.2)	20 (47.6)	27 (33.3)	0.122
Diabetes *n* (%)	37 (30.1)	12 (38.6)	25 (30.9)	0.838
Medical history of hypertension *n* (%)	79 (64.2)	31 (73.8)	48 (58.3)	0.119
Median Charlson score (IQR)	6 (4–8)	6 (5–8)	5 (4–7)	0.1228
Smokers *n* (%)	16 (13)	3 (7.1)	13 (16.1)	0.258
High alcohol consumption *n* (%)	19 (15.5)	4 (9.5)	15 (18.5)	0.293
Mean serum albumin (g/l) (std) ***	23.7 (6.7)	22.7 (6.3)	24.2 (7)	0.3669
PPI *n* (%)	53 (43.1)	22 (52.4)	31 (38.3)	0.179
NSAID *n* (%)	9 (7.3)	3 (7.1)	6 (7.4)	1.000
RAAS inhibitors *n* (%)	45 (36.6)	20 (47.6)	25 (30.9)	0.078
Loop diuretics *n* (%)	45 (36.6)	22 (52.4)	23 (28.4)	0.011
Non-loop diuretics *n* (%)	30 (24.4)	15 (35.7)	15 (18.5)	0.046
Statins *n* (%)	38 (30.9)	13 (30.9)	25 (30.9)	1.000
Vancomycin *n* (%)	26 (21.1)	13 (30.9)	13 (16.1)	0.065
Aminoglycosides *n* (%)	69 (56.1)	28 (66.7)	41 (50.6)	0.125
Other penicillins *n* (%)	45 (36.6)	15 (35.7)	30 (37)	1.000
Iodinated contrast media injection (%)	37 (30.1)	15 (33.3)	23 (28.4)	0.679
Hypotension	16 (13)	9 (21.4)	7 (8.6)	0.054
Infection *n* (%)				0.001
Endocarditis	30 (24.4)	18 (42.9)	12 (14.8)	
Other infections	93 (75.6)	24 (57.1)	69 (85.2)	
Bacteremia	47 (38.2)	13 (30.9)	34 (42)	
Bones infection	31 (25.2)	9 (21.4)	22 (27.2)	
Catheter blood stream infection	11 (8.94)	1 (2.4)	10 (12.3)	
Urinary tract infection	4 (3.3)	1 (2.4)	3 (3.7)	
Median dose g per day (min-max) *	10 (3–16)	12 (3–16)	10 (3–12)	0.4168
Method of administration *n* (%)				0.550
IV	120 (97.6)	42 (100)	78 (96.3)	
Oral	3 (2.4)	0	3 (3.7)	
Infusion type *n* (%)				0.329
Continuous	11 (8.9)	2 (4.8)	9 (11.1)	
Intermittent	112 (91.1)	40 (95.2)	72 (88.9)	
Median days of treatment (IQR) (min-max) *	9 (5–14) (2–45)	7 (3–13) (2–45)	10 (6–15) (2–45)	0.0675
Median days of hospital stay (IQR) (min-max) *	21 (5–35) (3–80)	27 (18.2) (3–80)	19 (15.7) (4–75)	0.0025
In-hospital death *n* (%)	24 (19.5)	12 (28.6)	12 (14.8)	0.093

IQR = Inter Quartile Range, std = standard deviation, * non-normally distributed variable, ** missing data for 11 patient, *** missing data for 46 patients. PPI: Proton Pump Inhibitors; NSAID: Nonsteroidal Anti-Inflammatory Drug. CKD: Chronic Kidney Disease.

**Table 2:** Characteristics of AKIs observed in patients included in the CLOX-AKI study, CH Le Mans from 1 July 2012 to 1 July 2019.

	AKI Group *n* = 42
Median creatinine at baseline μmol/L (IQR) (min–max)	93.5 (73–112) (45–183)
Median peak creatinine μmol/L (IQR) (min–max)	221 (150–332) (95–1006)
Dialysis *n* (%)	6 (14.3%)
Encephalopathy *n* (%)	4 (9.5%)
Skin reaction *n* (%)	4 (9.5%)
Hepatitis *n* (%)	13 (32.5%)
Eosinophilia *n* (%)	4 (9.5%)
Median proteinuria g/L (IQR) (min–max) *	0.52 (0.2–0.8) (0–4.34)
Median PCR g/g (IQR) (min–max) **	0.64 (0.38–2) (0–5.31)
Hematuria *n* (%) ***	9 (27.3%)
Leukocyturia *n* (%) ****	18 (56.3%)
Median creatinine at hospital discharge (IQR) (min–max)	140 (104–210) (49–429)
Median creatinine at M3 (IQR) (min–max) *****	102 (74–163) (62–230)

* 16 missing data ** 17 missing data *** 9 missing data **** 10 missing data ***** 26 missing data. PCR: Proteinuria/Creatininuria Ratio. M3: 3 months after discharge.

**Table 3:** Univariate analysis of factors associated with AKI in the CLOX-AKI study (only factors included in the multivariate analysis are presented).

Factors Studied	Crude OR	(95% CI) *	*p*
Age			0.0017
≤75		ref.	
>75	3.42	(1.55–7.54)	
CKD			0.124
No		ref	
Yes	1.82	(0.85–3.89)	
RAAS inhibitors			0.0691
No		ref.	
Yes	2.04	(0.95–4.39)	
Loop diuretics			0.0093
No		ref.	
Yes	2.77	(1.28–6.02)	
Non-loop diuretics			0.0383
No		ref.	
Yes	2.44	(1.05–5.69)	
Vancomycin			0.0595
No		ref.	
Yes	2.34	(0.97–5.67)	
Aminoglycosides			0.0866
No		ref.	
Yes	1.95	(0.90–4.24)	
Infection			0.0008
Other infections		ref.	
Endocarditis	4.31	(1.81–10.25)	
Hypotension			0.0516
No		ref.	
Yes	2.88	(0.99–8.40)	

* CI95% = 95% confidence interval.

**Table 4:** Multivariate analysis of factors associated with AKI in the CLOX-AKI study (final model).

Factors Studied	Adjusted OR	(95% CI) *	*p*
Age			0.002
≤75		ref.	
>75	4.38	(1.73–11.07)	
CKD			0.693
No		ref.	
Yes	0.82	(0.30–2.22)	
RAAS inhibitors	NS		
Loop diuretics			0.036
No		ref.	
Yes	2.94	(1.07–8.04)	
Non-loop diuretics			0.027
No		ref.	
Yes	3.05	(1.13–8.19)	
Vancomycin	NS		
Aminoglycosides	NS		
Infection			0.012
Other infections		ref.	
Endocarditis	3.42	(1.31–8.94)	
Hypotension	NS		

NS Not Significant. * CI95% = 95% confidence interval.

## Data Availability

The data presented in this study are available on reasonable request from the corresponding author. The data are not publicly available due to privacy.

## Supplementary Materials

The following are available online at https://www.mdpi.com/article/10.3390/jcm10122603/s1, Figure S1. Residuals of the multiple regression; Figure S2. Discrimination of the multiple regression.
