# Supplementary material for: Incidence and Risk Factors for Acute Kidney Injury during the Treatment of Methicillin-Sensitive Staphylococcus aureus Infections with Cloxacillin Based Antibiotic Regimens: A French Retrospective Study
Source: J Clin Med. 2021 Jun 12;10(12):2603. doi: 10.3390/jcm10122603 (PMC8231553; doi:10.3390/jcm10122603)
Supplement: Supplementary file 1 [file jcm-10-02603-s001.zip › jcm-1234677-supplementary.pdf]

Supplementary material

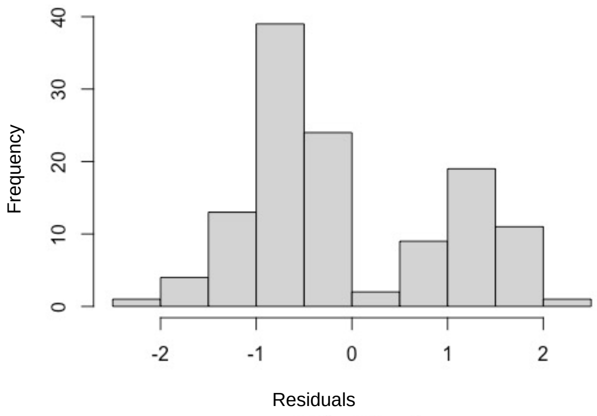

Figure 1. Residuals of the multiple regression. Calibration of the model was good with a Hosmer-Lemeshow chi2 of 9.30 (p= 0.1575).

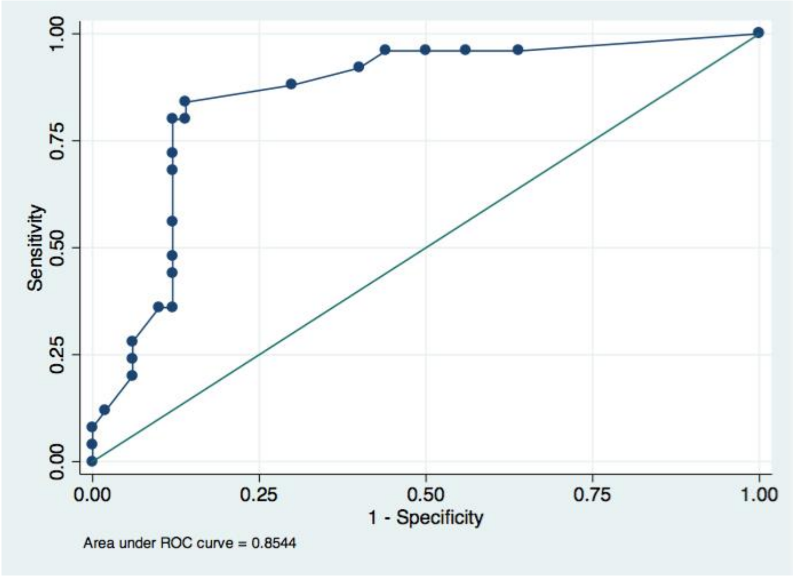

Figure S2. Discrimination of the multiple regression.
